# Supplementary material for: Reconciling Mining with the Conservation of Cave Biodiversity: A Quantitative Baseline to Help Establish Conservation Priorities
Source: PLoS One. 2016 Dec 20;11(12):e0168348. doi: 10.1371/journal.pone.0168348 (PMC5173368; doi:10.1371/journal.pone.0168348)
Supplement: S1 Dataset — (ZIP) [file pone.0168348.s002.zip › Taxa/Serra Norte/SN_2013/N4WS/Lista N4WS-69.pdf]

CAVIDADE N4WS-0069

Classe	Ordem	Fam/Outros	Gên/Outros	Espécie	S-U	U-S	Zon.
Arachnida	Amblypygi	Phrynidae	*Heterophrynus*	*longicornis*	3	1	P
Arachnida	Araneae	Ctenidae		jovem	1		P
Arachnida	Araneae	Drymusidae		jovem	X		E
Arachnida	Araneae	Oonopidae		jovem		X	P
Arachnida	Araneae	Oonopidae		jovem	X		P
Arachnida	Araneae	Theraphosidae		jovem	1	1	E
Arachnida	Araneae	Theridiosomatidae	*Plato*	sp.1		X	E
Diplopoda	Spirostreptida	Pseudonannolenidae	*Pseudonannolene*	sp.1		1	P
Entognatha	Collembola	Entomobryidae		sp.6		X	E
Entognatha	Collembola	Paronellidae		sp.1	X		E
Entognatha	Collembola	Paronellidae		sp.4	X		P
Insecta	Diptera	Psychodidae	*Evandromyia*	*monstruo*		X	P
Insecta	Diptera	Psychodidae	*Pintomyia*	*gruta*	X		E/P
Insecta	Diptera	Psychodidae	*Sciopemyia*	*sordellii*		X	P
Insecta	Heteroptera	Schizopteridae	Schizopterinae	sp.1		X	E
Insecta	Homoptera	Cercopidae		jovem	X		E
Insecta	Hymenoptera	Formicidae	*Camponotus*	sp.1	X	X	E/P
Insecta	Hymenoptera	Formicidae	*Pachycondyla*	*striata*		1	E
Insecta	Hymenoptera	Formicidae	*Solenopsis*	sp.1	X		P
Insecta	Isoptera	Termitidae	*Nasutitermes*	sp.	X		E/P
Insecta	Lepidoptera	Noctuoidea		sp.1	2		P
Insecta	Lepidoptera	Tineoidea		sp.3		X	P
Insecta	Orthoptera	Phalangopsidae	*Paraclodes*	sp.	8		E
Insecta	Orthoptera	Phalangopsidae	*Phalangopsis*	sp.	6	4	P
Insecta	Psocoptera	Ptiloneuridae	*Ptiloneura*	sp.1		X	E
Amphibia	Anura	Strabomantidae	*Pristimantis*	cf. *fenestratus*	3	1	E/P
Mammalia	Chiroptera	Phyllostomidae	*Carollia*	*perspicillata*		6	
